# Rapid Progression of Invasive Listeria monocytogenes Infection in a Patient With Cirrhosis and Primary Sclerosing Cholangitis on Ustekinumab

**DOI:** 10.7759/cureus.58116

**Published:** 2024-04-12

**Authors:** Laura Budvytyte, Mariah Schroeder, Erin Graf, James J Vaillant

**Affiliations:** 1 Department of Laboratory Medicine and Pathology, Mayo Clinic Alix School of Medicine, Scottsdale, USA; 2 Division of Clinical Microbiology, Department of Laboratory Medicine and Pathology, Mayo Clinic, Phoenix, USA; 3 Division of Clinical Microbiology, Department of Laboratory Medicine and Pathology, Mayo Clinic, Rochester, USA

**Keywords:** ustekinumab, cirrhosis, meningitis, peritonitis, listeriosis, listeria monocytogenes

## Abstract

We present the case of a 62-year-old immunocompromised man with ulcerative colitis, primary sclerosing cholangitis, and cirrhosis treated with azathioprine and ustekinumab who quickly developed invasive *Listeria monocytogenes* infection after incidental identification on routine paracentesis. The infection rapidly progressed from bacterial peritonitis to bacteremia and meningitis within three days. Treatment with ampicillin and trimethoprim/sulfamethoxazole was successful. We highlight the increased risk of invasive listeriosis in immunocompromised individuals, including those on biologic therapies, and the importance of considering *Listeria* as a pathogen from sterile sites even in asymptomatic patients.

## Introduction

*Listeria monocytogenes* is an aerobic Gram-positive bacillus transmitted via the gastrointestinal tract. Exposure to *L. monocytogenes* can result from the consumption of contaminated foods, contact with contaminated surfaces, or vertical transmission. Once infected, patients can have a broad range of clinical presentations, ranging from mild gastroenteritis to severe, invasive disease [1]. Immunocompromised individuals, pregnant women, and those at extremes of age are at the greatest risk for severe illness. Increased risk has been associated with biologic immunotherapies [2]. Here, we present a case of rapid progression to invasive neurolisteriosis in an immunocompromised patient with a complex medical history including primary sclerosing cholangitis (PSC), cirrhosis, and ulcerative colitis managed with azathioprine and ustekinumab. This case underscores the importance of maintaining a heightened clinical suspicion for atypical presentations of *L. monocytogenes* infections in immunocompromised patients to enable timely diagnosis and mitigate associated morbidity and mortality.

## Case presentation

**Figure 1:**
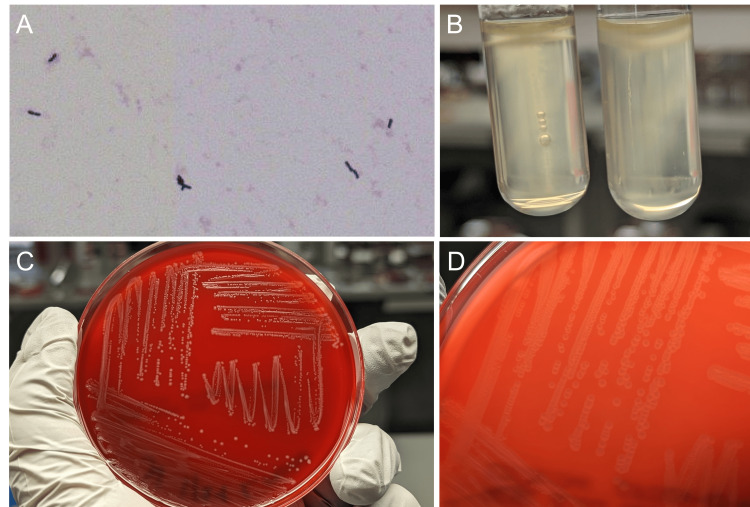
(A) Microscopic Gram stain morphology from the positive blood culture bottle that was inoculated with the patient’s peritoneal fluid. (B) Motility after incubation at 35°C (left) and at room temperature (right). (C) Macroscopic morphology after subculture on sheep blood agar. (D) Narrow zone of β-hemolysis after subculture on sheep blood agar.

A 62-year-old male presented to the ED with a three-day history of nausea and diarrhea. His past medical history was significant for ulcerative colitis treated with azathioprine 100 mg daily and ustekinumab 90 mg subcutaneously every eight weeks complicated by PSC and cirrhosis. A routine therapeutic paracentesis had been performed three days prior, at which time, an analysis of the fluid showed a total nucleated cell count (TNC) of 198 cells/µL with 8% neutrophils. The peritoneal fluid was sent for routine bacterial cultures and was plated onto sheep blood and chocolate agars and inoculated into an aerobic blood culture bottle (BACTEC, Becton Dickinson, Franklin Lakes, New Jersey, United States). The solid media showed no growth after three days of incubation; however, the blood culture bottle flagged positive on the automated system at 25 hours and the Gram stain of the broth revealed small Gram-positive rods (Figure 1A). After subculture onto sheep blood agar, small, grey β-hemolytic colonies were visible after 18 hours (Figures 1C, 1D). 

The bacteria were identified as* L. monocytogenes* via matrix-assisted laser desorption ionization-time of flight mass spectrometry (MALDI-TOF MS) (MALDI Biotyper, Bruker Daltonics, Inc., Billerica, Massachusetts, United States) and immediately reported to the ordering physician. At the time of the paracentesis, the patient’s only symptom was nausea, and the growth of *Listeria* was initially clinically regarded as a potential contamination. Upon presentation to the ED three days later, he was febrile and tachycardic. His abdomen was non-tender, soft, and protuberant. The laboratory evaluation showed hyponatremia, leukocytosis of 11.9×109 cells/L with neutrophilic predominance, and elevated lactate. Blood cultures were collected and a paracentesis was performed. The peritoneal fluid analysis showed a TNC of 346 cells/µL with 79% neutrophils, consistent with spontaneous bacterial peritonitis (SBP). Gram stain of the fluid was negative for any organisms. The patient was admitted to the hospital and administered piperacillin-tazobactam 3.375 g IV every six hours for sepsis secondary to SBP. 

After incubation for 17 hours, cultures from both the blood and peritoneal fluid grew *L. monocytogenes*. The patient became tachycardic, tachypneic, febrile, and dyspneic, and was transferred to the ICU. Antimicrobials were switched to ampicillin 2 g IV every four hours and trimethoprim/sulfamethoxazole (TMP/SXT) 400 mg TMP component IV every eight hours for directed treatment of invasive listeriosis. A transthoracic echocardiogram showed no evidence of infective endocarditis. The following day, the patient developed neck rigidity and headache. A lumbar puncture revealed a hazy appearance of the CSF with a TNC of 2125 cells/µL with 87% neutrophils. Multiplex meningitis/encephalitis polymerase chain reaction (PCR) panel and bacterial cultures of the CSF were positive for *L. monocytogenes*. On hospital day five, antimicrobial therapy was consolidated to TMP/SXT 1600-320 mg orally three times daily and penicillin G 24 million units IV daily. 

A lumbar puncture was repeated on hospital day seven due to intermittent headaches. TNC of the CSF had declined to 367 cells/µL. Gram stain, bacterial culture, and multiplex meningitis/encephalitis PCR panel of the CSF were negative. On day 11, the patient was stable and discharged from the hospital to continue penicillin G and TMP/SXT for a total of eight weeks, followed by chronic antibiotic suppression with TMP/SXT thereafter, given his ongoing immunocompromised status. The patient did not exhibit signs of disease relapse post-treatment and continued routine paracenteses for PSC without complications.

## Discussion

*L. monocytogenes* is a ubiquitous Gram-positive bacillus and is the causative agent of listeriosis. Of the 26 described species in the genus, *L. monocytogenes* is the most frequent cause of human infections [3]. It is a facultative anaerobe that grows well on sheep blood agar between 30°C and 37°C and produces a small zone of β-hemolysis, which may be obscured by the colony (Figure 1C). Classical biochemical features include catalase positivity, tumbling motility on wet mount, motility at room temperature with an umbrella‐like growth pattern (Figure 1B), hippurate hydrolysis, and esculin hydrolysis. These features are adequate to provide presumptive identification in clinical laboratories, although definitive identification can be achieved with MALDI‐TOF MS or commercial biochemical testing kits [3,4]. *L. monocytogenes* can also be detected directly from positive blood culture broth or CSF using FDA-cleared nucleic acid amplification test panels. Although gastrointestinal infections are common, there are currently no guidelines nor available tests for* L. monocytogenes* in stool specimens.

*Listeria* is typically acquired via the gastrointestinal tract and has a broad range of clinical presentations from self-limiting gastroenteritis to invasive systemic infections like bacteremia and meningitis. Cases may occur sporadically, or result from foodborne transmission from contaminated cheese, processed meats, and produce. Individuals at greatest risk for invasive forms of the disease include pregnant women, neonates, and immunocompromised individuals [1]. Among invasive infections, bacteremia is the most common, followed by neurolisteriosis [5]. Focal infections, such as peritonitis, are uncommon. Despite its rarity, *Listeria* has been well-described as a cause of SBP in cirrhotic patients and secondary peritonitis in patients undergoing peritoneal dialysis [6-12]. Upon entering the gastrointestinal tract, *L. monocytogenes* invades the intestinal epithelium into the lamina propria. From there, it disseminates from the lymphatics into the bloodstream and travels toward the liver and the spleen. Once in the bloodstream, it can cross the blood-brain barrier leading to neurolisteriosis [13]. *L. monocytogenes *has been shown to evade host defense factors by disrupting the phagosomal membrane using virulence factors of listeriolysin O and phospholipases and escaping the internalized vacuole. It may then use actin motility or receptor-mediated endocytosis to enter nearby cells [13,14]. 

The treatment of choice for invasive listeriosis is IV ampicillin or penicillin, typically in conjunction with gentamicin. Alternative therapies include TMP/SXT or meropenem in cases of severe penicillin allergy or other contraindications to first-line agents. Bacteremia is generally treated for at least two weeks, while central nervous system (CNS) disease may be treated for three weeks or longer, with duration individualized based on the severity of illness and immunodeficiency. The treatment of gastrointestinal infection is usually not necessary in immunocompetent hosts. Antibiotic susceptibility testing of *Listeria* isolates is not routinely performed due to predictable susceptibility to penicillin and ampicillin, and the Clinical and Laboratory Standards Institute (CLSI) provides interpretive breakpoint criteria only for the susceptible category [15]. Furthermore, laboratory workers should avoid unnecessary manipulation of *L. monocytogenes,* which may pose a risk for laboratory transmission.

United States state public health laboratories and the CDC actively surveil for *L. monocytogenes* cases and perform molecular analyses on isolates to identify outbreaks. In 2022, the CDC reported 136 cases with 128 hospitalizations and 30 deaths (22% case fatality rate). The incidence of listeriosis in the United States has remained stable between 2016 and 2022 [16]. Foodborne outbreaks are identified several times per year with associated product recalls. The case patient did not have an identifiable source for his exposure but was interviewed by public health authorities. The risk of *Listeria* infection can be reduced by safe food handling, and this is especially important for immunocompromised individuals. Such practices include frequent handwashing when preparing food, and avoiding consumption of unpasteurized milk or juices, cheeses made using unpasteurized milk, and food prepared by any individuals with recent diarrheal illness [17]. 

Immunocompromised states have been associated with invasive *Listeria* infections. These include cirrhosis, diabetes mellitus, end-stage renal disease, inflammatory bowel disease, autoimmune disease, malignancies, hematopoietic or solid-organ transplant, primary or acquired immunodeficiencies, and immunosuppressive therapies [5,14]. Among the latter, there are numerous reports of listeriosis in patients receiving biologic therapies [2]. A review of 266 cases of biologic therapy-associated *Listeria* infections from January 2000 to September 2011 found that 77.1% were associated with infliximab, 11.7% with etanercept, 9.8% with adalimumab, 4.1% with rituximab, 0.4% with abatacept, and 0.4% with golimumab. About 73% of the cases were associated with concomitant corticosteroid or methotrexate therapy [2]. Indeed, listeriosis appears to be more common among individuals receiving biologic therapies, although the precise risk that specific biologic agents confer remains unclear due to limitations in retrospective study designs.

At least one other case of neurolisteriosis has been reported in a patient receiving ustekinumab [18]. A patient taking ustekinumab for plaque psoriasis was diagnosed with *Listeria* meningitis from a lumbar puncture. Treatment with seven days of gentamicin and four weeks of ampicillin led to the resolution of symptoms. Ustekinumab is an inhibitor of IL-12 and IL-23, which are primarily produced by macrophages and dendritic cells. These cytokines have a significant role in the activity of natural killer cells, the production of interferon-γ, and Th1 polarization of CD4+ T cells [18,19]. The impairment of these functions may theoretically increase vulnerability to intracellular pathogens [19]. Our patient was asymptomatic at the time of initial identification of *Listeria* in the peritoneal fluid and progressed to bacteremia and meningitis within 72 hours. It is likely that impairment in inflammatory cell recruitment to the site of infection, as evidenced by low nucleated cell count in the peritoneal fluid, allowed relatively unchecked bacterial growth and progression to disseminated disease in a short period. A high index of clinical suspicion for infection should always be maintained in immunocompromised hosts who may otherwise not mount a robust inflammatory response or present with typical symptoms. 

## Conclusions

This case illustrates the potential for rapid progression of invasive listeriosis in patients with multiple risk factors receiving biologic therapies. The identification of *L. monocytogenes* from any source should always be considered pathogenic, particularly from a sterile site such as the peritoneal cavity. Timely diagnosis and treatment are critical to prevent rapid progression to invasive listeriosis. Future studies with larger populations of immunocompromised individuals will further clarify the relative risks of various specific immunosuppressive agents. 
